# The Common Element Effect of Abstract-to-Abstract Mapping in Language Processing

**DOI:** 10.3389/fpsyg.2016.01623

**Published:** 2016-10-24

**Authors:** Xuqian Chen, Guixiang Wang, Yuchan Liang

**Affiliations:** ^1^Center for the Study of Applied Psychology, Guangdong Key Laboratory of Mental Health and Cognitive Science, School of Psychology, South China Normal UniversityGuangzhou, China; ^2^Division of Student Affairs, Psychological Health Center, Guangzhou Academy of Fine ArtsGuangzhou, China

**Keywords:** common elements, abstract action, time, length, mapping

## Abstract

Since the 1990s, there has been much discussion about how concepts are learned and processed. Many researchers believe that the experienced bodily states (i.e., embodied experiences) should be an important factor that affects concepts’ learning and use, and metaphorical mappings between abstract concepts, such as TIME and POWER, and concrete concepts, such as SPATIAL ORIENTATION, STRUCTURED EXPERIENCEs, etc., suggest the abstract-concrete concepts’ connections. In most of the recent literature, we can find common elements (e.g., concrete concepts) shared by different abstract-concrete metaphorical expressions. Therefore, we assumed that mappings might also be found between two abstract concepts that share common elements, though they have no symbolic connections. In the present study, two lexical decision tasks were arranged and the priming effect between TIME and ABSTRACT ACTIONs was used as an index to test our hypothesis. Results showed a robust priming effect when a target verb and its prime belonged to the same duration type (TIME consistent condition). These findings suggest that mapping between concepts was affected by common elements. We propose a dynamic model in which mappings between concepts are influenced by common elements, including symbolic or embodied information. What kind of elements (linguistic or embodied) can be used would depend on how difficult it is for a concept to be learned or accessed.

## Introduction

What is LOVE? We cannot see it or touch it, but can only feel it or experience it. “An individual’s concept of LOVE depends on both his or her own experiences plus the metaphorical concepts for LOVE provided by the culture” (Lakoff and Johnson, 1980, p. 206). Then what kind of information can people get from their experience? Using the concept LOVE as an example, we never really experience what love (i.e., abstract concept) is, but learn what we will do (i.e., concrete concepts and the relevant related experiences, such as KISS, ARGUE, FIGHT, etc.), when we are in LOVE. By doing so, we build up the concept LOVE. As a consequence, researchers are interested in how people think about things they have never experienced, and the notion of embodiment has been offered as a possible solution to this problem. The main idea underlying embodiment is that higher cognitive processes rely heavily on the brain’s modality-specific systems and on actual bodily states (Niedenthal et al., 2005). According to the strong embodied approach, researchers believe that cognitive processes such as concept learning and concept use involve partial reactivations of the sensory-motor states (i.e., simulation) that occur during experience with the world (Glenberg, 1997; Niedenthal et al., 2009).

In most of the recent literatures, the view of embodiment was discussed based on the idea of conceptual metaphors (Santiago et al., 2011). The embodiment view of abstract concepts has its origins in conceptual metaphor theory, which emerged within cognitive linguistics with a stronger focus on representation than on processing (Lakoff and Johnson, 1980, 1999; Johnson, 1987; Gibbs and O’Brien, 1990). For example, according to what was in our conceptual schema built by experience, we have some metaphorical sayings for LOVE, such as LOVE IS A JOURNEY or LOVE IS WAR. When people use the “A is B” metaphor, A is considered as the target domain while B is the source domain (Lakoff, 1993). The source domain (e.g., JOURNEY) lays the foundation for the concept, which in its turn forms mappings, or conceptual metaphors. The conceptual metaphor will further provide a whole number of linguistic expressions, or as we might call them linguistic metaphors, that finally deliver the idea to the target domain (e.g., LOVE). In other words, “the human conceptual apparatus may look like the Empire State Building: a rock-solid structure where upper (more abstract) floors are supported by lower (more concrete) floors” (Santiago et al., 2011, p. 43).

However, it is still not clear whether embodied experience was involved in mappings between these abstract and concrete concepts. Some researchers have argued that sensorimotor system activation or experiences during language processing are not as necessary as those mentioned by the strong embodied approach for comprehension (for reviews, see Sakreida et al., 2013). Using fMRI as an index, Sakreida et al. (2013) claimed that concrete word processing relies more on the sensorimotor system, whereas abstract word processing relies more on the linguistic system, though the sensorimotor neural network was found to be engaged in both concrete and abstract language contents in their study. These processing differences between concrete and abstract concepts can be also captured when the processing of phrases is discussed (Scorolli et al., 2012). According to the strong embodied approach and its opposite view, we believe that there might be a possible combination: Reactivations of the sensory-motor states, or so-called sensory-motor states in Sakreida et al.s’ (2013) study, are indirectly involved in abstract concepts’ learning and use, while the concrete concepts act as mediators.

By analyzing discussions about the metaphorical expressions, we found that metaphorical concepts were always abstract concepts (i.e., difficult to be experienced), whereas non-metaphorical ones were more concrete (i.e., easy to be experienced). In the conceptual metaphor theory (Lakoff and Johnson, 1980), non-metaphorical concepts include at least (1) spatial orientations (e.g., UP–DOWN, IN–OUT, NEAR–FAR, and FRONT–BACK), (2) ontological concepts arising in physical experience (e.g., ENTITY, SUBSTANCE, CONTAINER, and PERSON), and (3) structured experiences and activities (e.g., EATING, MOVING, TRANSFERRING OBJECTS FROM PLACE TO PLACE). Evidence in support of this theory came from research on spatial metaphors, such as the POWER and BIG–SMALL metaphor (Freedman, 1979; Lakoff and Johnson, 1980, 1999; Schubert et al., 2009; Yap et al., 2013), POWER and UP–DOWN metaphor (Schubert, 2005; Meier et al., 2007; Zanolie et al., 2012), TIME and UP–DOWN/LEFT–RIGHT metaphor (Boroditsky, 2000; Fuhrman and Boroditsky, 2007; Santiago et al., 2007; Liu and Zhang, 2009a,b; Ouellet et al., 2009), TIME and LONG–SHORT metaphor (Casasanto and Boroditsky, 2008; Zäch and Brugger, 2008; Chen and Zhang, 2011), and EMOTION and UP–DOWN metaphor (Stefanucci and Storbeck, 2009; Lü and Lu, 2013). Evidence also came from metaphors of structured experiences and activities, such as the SOCIAL EMOTION and WARM–COLD metaphor (Williams and Bargh, 2008; IJzerman and Semin, 2010; Bargh and Shalev, 2012; IJzerman et al., 2012) and EMOTION and FACIAL EXPRESSION metaphor (Strack et al., 1988).

As a consequence, learning an abstract concept might be directly facilitated by a metaphorical expression, in which there is embodied mapping between bodily states and the concrete concepts (i.e., “embodied schema” in short in the current study). For example, when people learn the abstract concept RELATIONSHIP and its metaphorical expression such as “We are CLOSE friends,” all embodied information about NEAR–FAR states could be activated and then affect the concept RELATIONSHIP learning. If this argument were true, the abstract-concrete mapping can be found in discussions in a more general way. Here, we further reflect on whether there is also mapping between two abstract concepts that share the same experience information according to their embodied schema, which was built by the relative concrete concepts and their sensorimotor system.

In language processing, especially based on the amodal symbols view (Burgess and Lund, 1997; Landauer and Dumais, 1997), robust priming effects have been widely found in conditions in which primes and targets share the same semantic or other common elements (Plaut, 1995; Bi et al., 1998; Sun and Wang, 2012; Chen et al., 2016). One of the typical examples is the DOCTOR–NURSE word pair, in which both words belong to the same semantic network in our mental lexicon. However, there is still a lack of research on whether similar priming effects can be found between primes and targets sharing common embodied information (e.g., similar experience). It is not surprising that some concepts, such as UP, can be used as the source domain in different metaphorical/embodiment expressions: MORE is UP, CONTROL is UP, GOOD is UP, RATIONAL is UP (Lakoff and Johnson, 1980). Therefore, there is common embodied information among different target domains (e.g., MORE, CONTROL, GOOD, and RATIONAL). By contrast, the same target domain can correspond to different source domains. For example, TIME, as an abstract concept, has several spatial embodied mappings, such as UP–DOWN, LEFT–RIGHT, FRONT–BACK, and LONG–SHORT (Casasanto and Boroditsky, 2008; Zäch and Brugger, 2008; Chen and Zhang, 2011), and some other valuable entailments (Lakoff and Johnson, 1980). There comes the possibility: If the same embodied schema is shared by two abstract concepts in our long-term memory, the schema might act as the common element and affect our concept processing.

To investigate this possibility, we chose two concepts—TIME and ABSTRACT ACTIONs— that they do not have directly symbolic connections, and more importantly, that both match the definition proposed by Lakoff and Johnson (1980): These two concepts are understood and structured not merely on their own terms, but also in terms of other concepts. Firstly, the perception of TIME is part of human experience. Based on the experience of the passage of time and anticipated duration, we make everyday decisions, as simple as either waiting for the elevator or taking the stairs (Wittmann and Paulus, 2008). However, “Time is a sort of river of passing events, and strong is its current” (Marcus Aurelius [translated version] Meditations, IV, 43, See Liverence and Scholl, 2012, p. 549). The very expression “the perception of time” invites objection because time is something different from events. When we have “the perception of time,” we indeed perceive embodied experiences such as SPATIAL DISTANCEs and other relations between objects (Le Poidevin, 2011). Therefore, we might take stairs to the second floor but wait for the elevator to the 20th. Distances (LONG/SHORT) are different between these two conditions. Secondly, compared with more concrete actions like KISS and STEP that can be seen and understood directly, JUDGE and THINK are more abstract in that they express mental processes, with no reference to a physical object (Scorolli et al., 2012). These actions might have no direct metaphorical mappings, but can be experienced as LONG or SHORT. These experiences are very important for ABSTRACT ACTIONs (Borghi and Cimatti, 2009, 2012), and they are always reflected in our daily expressions, in a somewhat complicated fashion.

For example, in the above elevator/stairs experiences of TIME, distances from the starting point to the goal for specific durations can be easily measured, but it is difficult to find a physically corresponding length for ABSTRACT ACTIONs. Then what could be the common element between TIME and ABSTRACT ACTIONs? In our daily expressions, connections between TIME and LENGTH (an hour is *longer* than a minute), and between ABSTRACT ACTION and LENGTH (it took me a *long* time to finish my homework) can be found. Therefore, no matter whether it was an experience of an hour or of fulfilling an action, these can be seen as events with starting and ending points, and the time (duration) for accomplishing these events can be perceived. As a consequence, LENGTH, specifically representing the experiences from the starting point to the ending point of the given event, can be considered as the common element shared by ABSTRACT ACTIONs and TIME. Therefore, concepts involving ABSTRACT ACTIONs (as indicated by the ratings made by participants in the present study) and TIME are suitable for the present research.

Based on the relationships between TIME and LENGTH (i.e., involving metaphorical expressions), and ABSTRACT ACTIONs and LENGTH (i.e., involving no metaphorical expressions but only experiences), it is assumed that there might be an “abstract-to-abstract” mapping with experience as the common element (i.e., “ABSTRACT ACTION-LENGTH-TIME”) during verb processing. In the present study, experience of TIME was manipulated by LENGTH-changing events (i.e., length-changing lines or duration-changing beeps). If this abstract-to-abstract mapping can be quickly activated under the priming task, verb processing should be facilitated by the TIME consistent condition. In other words, the goal of this research was to test whether mappings between two abstract concepts could be connected by their common concrete concept in word processing.

This hypothesis was tested in two experiments. In Experiment 1, abstract words with different DURATION mappings were primed by two kinds of length-changing lines, so that the metaphorical priming effect could be detected under a visual condition. For reasons of experimental rigor, Experiment 2 was arranged to be identical to Experiment 1 except the primes used in Experiment 1 were changed in order to offset any tau effect. The tau effect is a spatial perceptual illusion that reveals that stimulus timing affects the perception of stimulus spacing: The greater the difference between the temporal intervals, the greater must be the spatial difference in the opposite sense in order to offset the tau effect (Helson and King, 1931). There are several explanations for this effect, and one of them is the constant velocity hypothesis (Jones and Huang, 1982), according to which the brain expects spatial intervals that would yield movement with constant velocity. Though we did not use temporal visual intervals, continuous lines might also cause this expectation. Because this so-called tau effect might be involved in Experiment 1, it is not quite clear whether the priming effect, if any, would be caused by the “ABSTRACT ACTION-TIME” mapping or by the “ABSTRACT ACTION-LENGTH (perception of LENGTH caused by the tau effect)” mapping. Thus, in Experiment 2, pure sound beeps with different durations were presented as the primes instead of the length-changing line, so that any influence of the tau effect could be reduced. The common property shared by two kinds of prime in the present study (a line in Experiment 1, a beep in Experiment 2) was that participants could perceive experience from the starting point to the ending point of the prime’s presentation.

## Experiment 1

### Method

#### Participants

Thirty-five right-handed Chinese native speakers took part in the present study (*M*_age_ = 21.25, *SD* = 2.07; 12 male). Participants gave informed consent before taking part in the experiment.

#### Materials

Eighty-five words (49 referring to long-time verbs, and 36 referring to short-time verbs) were selected from the Dictionary of Modern Chinese Language (Institute of Language, 2008), and 60 students who did not take part in the main study completed the following rating questionnaires (20 students for each). Firstly, abstractive degree (abstractness) of candidates was rated on a 7-point scale (1 = concrete action, 7 = abstract verb). In addition, most of the Chinese words were multi-category words such that verbs and nouns were sometimes difficult to identify. Therefore, all candidate words were also rated according to their typical category on a 7-point scale (category rating, 1 = absolute noun, 7 = absolute verb), and only words whose means of the abstractive ratings and category ratings were higher than 5 were chosen for use in the experiment. All these chosen candidates were then rated according to their typical perception of duration on a 7-point scale (1 = such an action can be done in a very short time, 7 = it takes a long time to finish such an action), and verbs with ratings under 2.5 represented short-time verbs whereas verbs with ratings over 5.5 represented long-time verbs. Finally, 46 target words, 23 referring to long-time abstract verbs (occur over a longer period of time, e.g., 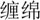 - lingering, 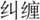 - tangle, etc.) and 23 short-time abstract verbs (occur over a shorter period of time, e.g., 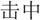 - hit, 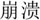 - breakdown, etc.) were selected for the present study. The two metaphorical durations (long vs. short) of the final selected words did not differ with respect to their familiarity, *t*(44) = 0.29, *p* > 0.05, or their category ratings, *t*(44) = 0.27, *p* > 0.05, but did differ significantly regarding ratings of their length, *t*(44) = 39.22, *p* < 0.001. Moreover, another 46 pseudo words were created as the filler probes so that the number of *Yes* and *No* answers could be balanced (details are in **Table 1**).

**Table 1:** Item characteristics for the final selected targets.

Variables controlled	Long-time verbs		Short-time verbs	
	*M*	*SD*	*M*	*SD*
Abstractness	5.50	0.33	5.52	0.32
Familiarity	5.64	0.45	5.68	0.47
Category rating	5.06	0.70	5.18	0.63
Typical perception of duration^∗∗∗^	5.83	0.32	2.03	0.33

*^∗∗∗^*p* < 0.001.*

In addition, two length-changing lines displayed as primes were created by *Macromedia Flash MX 2004*. One line was differentiated from the other by its duration. Duration from the starting point to completion of the final line (P-L duration) was 2000 ms for the long-time prime (10 visual-pictures per second), whereas the P-L duration for the short-time prime was 500 ms (40 visual-pictures per second). Therefore, both of these lines were visually of the same length when in the final position.

#### Apparatus and Procedure

**FIGURE 1:**
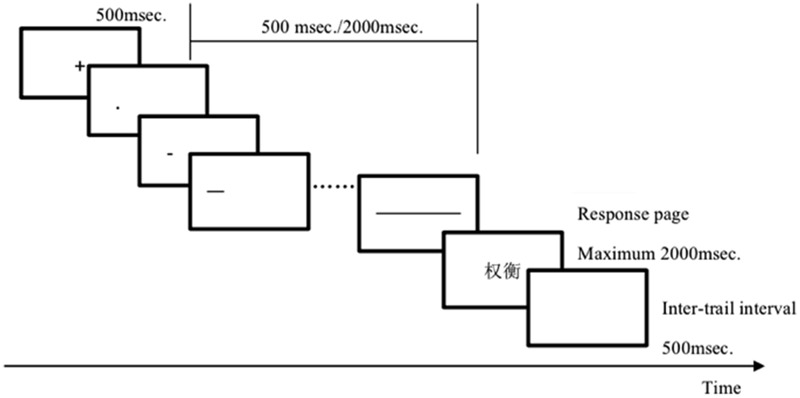
**Example trial procedure in Experiment 1.** In Experiment 2, pure sound beeps with different durations were displayed instead of the length-changing line.

A priming lexical decision task without feedback was used. The experimental software E-Prime presented the stimuli and recorded reaction times. Control files were constructed to display stimuli on a 17-inch IBM (9512-AB1) monitor (screen resolution: 1024 pixels × 768 pixels). Participants were tested individually in a sound-proof room. All priming lines were started from the left (*X*: 25% *Y*: 50%) of the screen to the right (*X*: 75% *Y*: 50%). Targets were presented in the center of the monitor. Each trial started with the presentation of a fixation cross for 500 ms. The fixation cross was replaced by a length-changing line (500 or 2000 ms), which was followed by the target display for maximum 2000 ms. Participants were asked to judge as quickly and as accurately as possible whether or not the target in each trial was a real word, and to press the corresponding button on a response box. The inter-trial interval was 500 ms (blank screen) and then the next trial started (see **Figure 1**). If the participant failed to respond in 2000 ms, the trial was terminated and recorded as an error. Participants received 10 practice trials, for which feedback was provided.

### Results and Discussion

**FIGURE 2:**
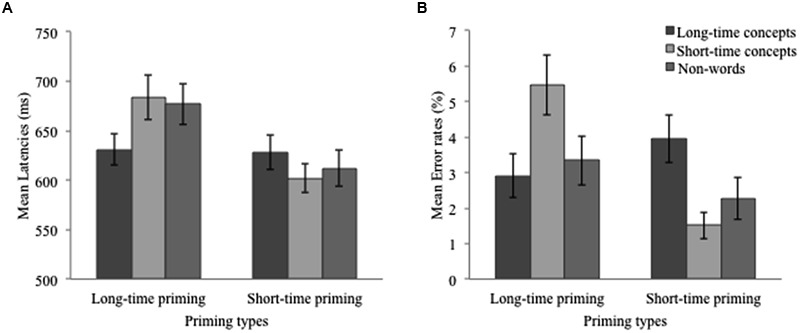
**(A)** Mean latencies and mean standard errors in Experiment 1. **(B)** Mean error rates and mean standard errors in Experiment 1.

Participants’ mean latencies (based on untrimmed correct responses) and error rates are summarized in **Table 2**, and graphically presented in **Figure 2**. Analyses of variance (2 × 2 ANOVAs) were conducted with priming type (duration for the priming lines: 500 ms vs. 2000 ms) and word type (different metaphorical time length for words) as within-subject factors and with latencies (ms) and error rates (%) as the dependent variables. In addition, as evidence showed that responses to real words and non-words might reflect different mechanisms during word accessing (Yang et al., 2006), latencies and error rates of non-words were submitted to separate *t*-tests.

**Table 2:** Means of latencies (SE) and error rates (SE) in Experiment 1.

	Long-time priming			Short-time priming		
	Long-time concepts	Short-time concepts	Non-words	Long-time concepts	Short-time concepts	Non-words
Latencies	631 (16)	684 (23)	677 (21)	628 (17)	602 (14)	612 (19)
Error rates	2.91 (0.62)	5.46 (0.83)	3.34 (0.69)	3.94 (0.67)	1.51 (0.39)	2.27 (0.59)

Results of the analyses on latency showed a significant difference between long-time priming (*M* = 657 ms) and short-time priming (*M* = 615 ms), *F*_1_(1,34) = 28.88, *p* < 0.01, η^2^ = 0.46, *F*_2_(1,22) = 23.27, *p* < 0.001, η^2^ = 0.51, and between long-time concepts (*M* = 629 ms) and short-time concepts (*M* = 643 ms), *F*_1_(1,34) = 4.30, *p* < 0.05, η^2^ = 0.11, *F*_2_(1,22) = 2.27, *p* > 0.10. Importantly, the interaction between priming type and word type reached significance, *F*_1_(1,34) = 16.53, *p* < 0.01, η^2^ = 0.33, *F*_2_(1,22) = 4.09, *p* = 0.056, η^2^ = 0.16. A further simple effect test showed that when primed by the long-time line, long-time concepts produced significantly smaller latency scores than short-time concepts, *F*_1_(1,34) = 14.09, *p* < 0.01, η^2^ = 0.29, *F*_2_(1,22) = 31.10, *p* < 0.001, η^2^ = 0.59, whereas when primed by the short-time line, long-time concepts produced significantly larger latency scores than short-time concepts, *F*_1_(1,34) = 9.08, *p* < 0.01, η^2^ = 0.21, *F*_2_(1,22) < 1, *ns*.

Similar analyses were done on error rates and results showed a significant difference between long-time priming and short-time priming, *F*_1_(1,34) = 5.56, *p* < 0.05, η^2^ = 0.14, *F*_2_(1,22) = 1.82, *p* > 0.10, but the main effect of word type was missing, *F*_1_(1,34) = 1.74, *p* > 0.05, *F*_2_(1,22) < 1, ns. Importantly, the interaction between priming type and word type also reached significance, *F*_1_(1,34) = 13.24, *p* < 0.01, η^2^ = 0.28, *F*_2_(1,22) = 5.71, *p* < 0.05, η^2^ = 0.21. A further simple effect test showed that long-time concepts primed by the long-time line produced significantly higher error rates than short-time concepts primed by the long-time line, *F*_1_(1,34) = 11.70, *p* < 0.01, η^2^ = 0.26, *F*_2_(1,22) = 5.96, *p* < 0.05, η^2^ = 0.21; by contrast, long-time concepts primed by the short-time line produced somewhat lower error rates than short-time concepts primed by the short-time line, *F*_1_(1,34) = 3.50, *p* = 0.07, η^2^ = 0.09, *F*_2_(1,22) = 1.21, *p* > 0.10.

The interaction between priming type and word type suggests that experience of TIME might act as mediator in the mapping between ABSTRACT ACTIONS and LENGTH. On the one hand, such findings are consistent with the view that TIME itself is not a property in the empirical world (Wittmann, 2009). When we talk about time (e.g., “an event lasted a long time,” “time flew by”), we use linguistic structures that refer to motion events and to locations and measures in space (Evans, 2004, see Wittmann, 2009). On the other hand, though VERBs used in the present study were somewhat abstractive, they are still relevant to corresponding motion events that people experience as time pressure (e.g., it takes seconds/minutes/hours to finish some ACTIONs) during concept learning (or everyday life). Thus, it was assumed that both TIME and ABSTRACT VERBs can be mapped to the same experience (i.e., experience of duration from starting point to ending point of the length-changing line) and interact with each other in the priming paradigm. The present results were in line with this hypothesis.

In addition, long-term priming trials showed disadvantages, no mater whether the following stimulus was a real word or a non-word. We believed that these findings could be explained by processing of internal analog (Moyer, 1973), which will be discussed later again with data of Experiment 2.

## Experiment 2

The hypothesis of the “ABSTRACT ACTION-LENGTH-TIME” mapping actually referred to an “abstract concept-to-abstract concept” mapping in the present research. However, though Experiment 1 showed that the LENGTH of TIME influences subsequent lexical decisions, the perception of time might become the perception of length of line under visual priming because of the tau effect. In what was probably the first experiment of this type, Benussi (1913, see Sarrazin et al., 2004) presented participants with three successive flashes of light defining two spatial and two temporal interstimulus intervals (ISIs). Distance judgments were found to vary as a function of the duration of the temporal ISIs (Sarrazin et al., 2004). Since then, many studies—involving the visual (Bill and Teft, 1969), auditory (Cohen et al., 1953), or kinesthetic (Helson and King, 1931; Lechelt and Bochert, 1977) modality—have shown that when two constant spatial ISIs are associated with variable temporal ISIs, distance is overestimated or underestimated in accordance with the temporal ISI. Though in Experiment 1 we did not have temporal ISIs, but continuous length-changing lines instead, expectation of movement with constant velocity from the starting point to the end of these lines still existed and there might be a similar tau effect involved in the results of Experiment 1. That is, participants might have perceived the changes of lines (concrete experience), thus affecting the following task. Therefore, instead of visual primes, beeps with different durations were used in Experiment 2 so that the abstractive property of priming could be more carefully manipulated.

### Method

#### Participants

Another 36 right-handed Chinese native speakers, who did not participate in Experiment 1, took part (*M*_age_ = 21.65, *SD* = 2.32; 13 male). Participants gave informed consent before taking part in the experiment.

#### Materials

Target words were identical to Experiment 1, but the visually length-changing lines were replaced by auditory pure sound (“beep”). A 500 ms beep was created by *Ulead Video Studio 12* for short-time priming while a 2000 ms beep was used for long-time priming.

#### Apparatus and Procedure

Experimental tasks were identical to Experiment 1, except that control files were constructed to play auditory primes through headphones (IRIVER AIRUNKE AE1) to participants, who were tested individually in a sound-proof room.

### Results and Discussion

**FIGURE 3:**
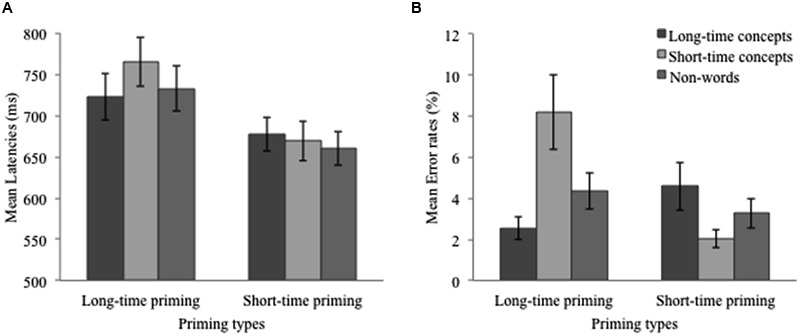
**(A)** Mean latencies and mean standard errors in Experiment 2. **(B)** Mean error rates and mean standard errors in Experiment 2.

Participants’ mean latencies (based on untrimmed correct responses) and error rates are summarized in **Table 3**, and graphically presented in **Figure 3**. Analyses of variance (2 × 2 ANOVAs) were conducted identical to those in Experiment 1.

**Table 3:** Means of latencies (SE) and error rates (SE) in Experiment 2.

	Long-time priming			Short-time priming		
	Long-time concepts	Short-time concepts	Non-words	Long-time concepts	Short-time concepts	Non-words
Latencies	723 (29)	765 (29)	733 (28)	677 (21)	669 (24)	660 (20)
Error rates	2.54 (0.54)	8.21 (1.83)	4.35 (0.87)	4.59 (1.17)	2.05 (0.45)	3.27 (0.71)

Results of the analyses on latency showed a significant difference between long-time priming (*M* = 744 ms) and short-time priming (*M* = 673 ms), *F*_1_(1,35) = 46.10, *p* < 0.01, η^2^ = 0.56, *F*_2_(1,22) = 30.91, *p* < 0.001, η^2^ = 0.58, and a marginally significant, but unreliable 17-ms, difference between long-time concepts (*M* = 717 ms) and short-time concepts (*M* = 700 ms), *F*_1_(1,35) = 3.81, *p* = 0.06, η^2^ = 0.10, *F*_2_(1,22) < 1, ns. Again, the interaction between priming type and word type reached significance, *F*_1_(1,35) = 10.17, *p* < 0.01, η^2^ = 0.23, *F*_2_(1,22) = 7.79, *p* < 0.05, η^2^ = 0.26. A further simple effect test showed that long-time concepts primed by the long-time beep produced significantly smaller latency scores than short-time concepts primed by the long-time beep, *F*_1_(1,35) = 24.78, *p* < 0.01, η^2^ = 0.42, *F*_2_(1,22) = 5.18, *p* < 0.05, η^2^ = 0.19, whereas a difference between long-time and short-time concepts primed by the short-time beep was not found, *F*_1_(1,35) = 0.30, *p* > 0.05, *F*_2_(1,22) = 4.67, *p* < 0.05, η^2^ = 1.75.

Results of error rates showed a significant difference between long-time priming and short-time priming, *F*_1_(1,35) = 4.78, *p* < 0.05, η^2^ = 0.12, *F*_2_(1,22) = 4.52, *p* < 0.05, η^2^ = 0.17, but the main effect of word type was again missing, *F*_1_(1,35) = 0.01, *p* > 0.10, *F*_2_(1,22) = 2.54, *p* > 0.10. Importantly, the interaction between priming type and word type reached significance, *F*_1_(1,35) = 16.28, *p* < 0.01, η^2^ = 0.32, *F*_2_(1,22) = 14.45, *p* < 0.01, η^2^ = 0.40. A further simple effect test showed that when primed by the long-time BEEP, long-time concepts produced significantly lower error rates than short-time concepts, *F*_1_(1,35) = 9.51, *p* < 0.01, η^2^ = 0.21, *F*_2_(1,22) = 14.82, *p* < 0.01, η^2^ = 0.40; however, when primed by the short-time BEEP, long-time concepts produced higher error rates than short-time concepts, *F*_1_(1,35) = 7.35, *p* < 0.05, η^2^ = 0.17, *F*_2_(1,22) = 3.61, *p* = 0.07, η^2^ = 0.14.

Under the auditory priming, the interaction between priming type and word type (i.e., difference between RTs of long-time and short-time words under the short-time priming condition) was a bit weakened but still apparent. These findings suggest that the priming effect was still a stable event without the concrete experience (e.g., expectation of movement of constant velocity in Experiment 1). According to the results of Experiments 1 and 2, the hypothesis of the “ABSTRACT ACTION-LENGTH-TIME” mapping was partly proved.

Moreover, similar to the pattern that was found in Experiment 1, long-term priming trials showed disadvantages on both real and non-words. These findings indicated that priming lines (Experiment 1) and beeps (Experiment 2) were actually processed in the current task, and they then affected the following lexical decision task. In one of Moyer’s (1973) studies, a size comparison task was designed to manipulate various size differences. Results showed that smaller size differences between target pairs lengthened RTs and increased error rates relative to larger size differences. Moyer believed that participants built “internal analogs” of the given stimuli and did “internal psychophysical (differences) judgment” in the task. The smaller the size differences were, the more difficult the judgment to be made.

Similarly, we believed that “internal analogs” of the given lines and beeps were also built during the present priming stage. However, different from research by Moyer (1973), processing of internal analogs in the present study was based on the “size (i.e., length)” of the given stimuli, rather than “size differences” of the stimulus pairs. According to opinion expressed in the literature (Kosslyn et al., 1978, 1979; Kosslyn, 1981), it took a longer time to process larger/longer internal analogs than shorter ones. Therefore, disadvantages were found with long-term priming trials, but not short-term priming trials.

In addition, although responses to real words and non-words should not be statistically compared (Yang et al., 2006), it was quite clear in both experiments that responses to non-words were slower than those to real words under the consistent condition but quicker than those to real words under the inconsistent condition. These results could be considered as further support for the hypothesis of the “ABSTRACT ACTION-LENGTH-TIME” mapping. That is, participants built the “length” mapping for the words (i.e., ABSTRACT ACTION) while they were building the “length” mapping for the primes (i.e., TIME). In the after-priming stage, processing should begin with accessing the word meaning and end up with the “length” consistency processing (between primes and words). Thus, there might be three kinds of effects in the present study: facilitation (e.g., under the length consistent condition), conflict (e.g., under the length inconsistent condition), or no interaction (e.g., under the non-word condition). Neither facilitation nor confliction could exist between primes and non-words because there were no “length” mappings for these words.

Therefore, the present results might be caused by building different “internal analogs” of the given primes (i.e., significant differences between responses under long- and short-term priming conditions), and also by the different consistencies of “length” mappings between primes and words (i.e., differences among consistent, inconsistent, and non-word conditions). However, these explanations were not convincing enough until further evidence was provided. Thus, the interactions between priming type and word type for the real words will be the main focus in the section “General Discussion.”

## General Discussion

Previous studies on embodied mappings have all focused on two concepts of which at least one was concrete, such as TIME and LENGTH (Casasanto and Boroditsky, 2008; Chen and Zhang, 2011), POWER and SIZE (Freedman, 1979; Lakoff and Johnson, 1980, 1999; Schubert et al., 2009; Yap et al., 2013), POWER and ORIENTATION (Schubert, 2005; Meier et al., 2007; Zanolie et al., 2012). These previous studies have focused on embodied mappings separately, but some so-called common elements (i.e., concrete concepts in more than one mapping) exist in some mappings (e.g., MORE is UP, CONTROL is UP, GOOD is UP, RATIONAL is UP, see Lakoff and Johnson, 1980). Therefore, it has been an open question whether there is mapping between two abstract concepts, when these concepts share common elements. This is the first study to test the possibility of this type of mapping. We found a robust priming effect when the primes and targets shared the same experience of length (TIME consistent condition), which was used as an index to test our hypothesis. Based on these findings, we propose a dynamic conceptual model in which mappings between concepts are influenced by common shared elements.

Firstly, our findings extend previous studies on embodiment theory. The most important extension is the idea that abstract concepts should be involved in our embodied schema, at least indirectly. To date, concrete concepts were considered the main part of embodied schema in the literature (Scorolli et al., 2012; Sakreida et al., 2013) and no mappings between two abstract concepts were discussed in relation to this schema. This is because researchers believed that “in general, abstract concepts are defined metaphorically in terms of concepts that are more concrete and more clearly structured on their own terms” (Lakoff and Johnson, 1980, p. 198). However, the embodied schema should be more complex. Findings from neuro-scientific studies on processing linguistic concreteness and abstractness showed that the sensorimotor neural network was engaged in processing abstract concepts but rather less so in processing concrete concepts (Scorolli et al., 2012; Sakreida et al., 2013). According to these findings, the embodied schema might be directly built by concrete concepts and their relative embodied simulations, and then the metaphorical connections between concrete and abstract concepts help build indirect connections between abstract concepts and the embodied simulations. In other words, if we think carefully about the relations among experience, language, and thoughts, we know we should build a more complex conceptual mapping: Those common concrete concepts and their relative embodied experiences help connect two abstract concepts, and the conceptual mappings should be built up not only with the connections between abstract and concrete concepts, but also with connections between abstract concepts.

Actually, this claim is also consistent with the Sapir-Whorf hypothesis, which pointed toward the possibility that grammatical differences reflect differences in the way that speakers of different languages perceive the world (for reviews, see Kay and Kempton, 1984). That is, the language we speak affects how we think about the world (Kay and Kempton, 1984; Hunt and Agnoli, 1991), and language is a powerful tool in shaping thought, especially habitual thought, about abstract domains (Boroditsky, 2001). Here, the notion of “thought” or “the way that we perceive the world” refers to the non-linguistic cognitive structures (Kay and Kempton, 1984), and, in the present study, precisely refers to the connections between concepts. Therefore, combining the embodiment theory (i.e., experience influences concrete concepts’ learning and use) and the Sapir-Whorf hypothesis (i.e., language/daily expression builds connections between concepts), we can infer a pathway for building up the embodied schema between abstract concepts and experiences: Experience + language (or metaphorical mappings between concrete and abstract concepts) thoughts. Using the connection among the concepts of ACTION, TIME, and LENGTH as an example: If LONG–SHORT experiences of events shape expressions such as “an hour is *longer* than a minute” and “it took me a *long* time to finish my homework,” then these expressions shape our thoughts, i.e., connections between concepts. The process of how people build up these expressions will be discussed again later. According to most of the recent literature, we believe these shaped thoughts (connections between concepts) finally affect the embodiment effects. Specifically in the present work, we argue that connections between two abstract concepts should exist in the conceptual mapping schema.

Next, we draw analogies between the amodal symbols view and embodiment theory according to the present findings, and we propose a dynamic system of word processing. When word processing was discussed in the literature under the amodal symbols view (Burgess and Lund, 1997; Landauer and Dumais, 1997), priming lexical decisions were always found to be affected by common elements between the prime and target in a symbolic system. These symbolic systems include a perceptual representation system (Tulving and Schacter, 1990; Marsolek et al., 1992), phonological system (Lukatela and Turvey, 1991, 1994; Lesch and Pollatsek, 1993; Harm and Seidenberg, 1999), orthographical system (Leck et al., 1995; Xu et al., 1999; Zhang and Weekes, 2009; Zhang et al., 2009; Zou et al., 2012; Chen et al., 2014), and semantic system (Plaut, 1995; Bi et al., 1998; Sun and Wang, 2012). When we learn a word, we also build up a complicated network for it. For example, when we learn the word SOFA, experience, such as seeing a picture of a sofa, or touching a real sofa, might help us to know what a sofa is. More importantly, we also learn SOFA is a kind of furniture and that it is usually put into a building, like chairs, tables, bed, etc., which can be used in our everyday life. Therefore, “a piece of furniture” and “we can use it every day” are common elements for sofa, chair, table, and bed. Some common elements are clear and direct (e.g., phonological and/or orthographical information such as the upper portion of the characters “
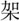
” and “
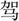
”, “word” and “work”), while some are more abstractive (e.g., semantic information). All this symbolic information will be encoded in our long-term memory, and affect our word processing.

Though symbolic information discussed under the amodal symbols view was not focused on under the traditional paradigm for discussing embodiment, researchers were actually also looking for some common elements. However, previous research has focused on common elements between two concrete concepts, and between concrete and abstract concepts, and until now there was still a lack of research on common elements between two abstract concepts. According to this point of view, we chose two concepts, TIME and ABSTRACT ACTIONs, which are both abstractive and have similar properties in the present study: Both TIME and ABSTRACT ACTIONs cannot be seen or touched; “experience” of these two concepts must be based on some other concrete concepts; it is never clear how “long” a time it is if it is discussed without specific events. At the same time, though people can feel how long an action lasts, this action cannot be physically measured as long or short, either. Importantly, these two concepts have some common elements: They both have long-short expression, but this long or short property cannot be directly measured. Findings in both experiments indicated that priming from the perception of time did influence the following word processing. There was mapping between the perception of TIME and ABSTRACT ACTIONs used in the present study, although they were both abstract. As the experimental manipulation in the present study, information about LENGTH might be the common element between two abstract concepts.

However, how do TIME, ABSTRACT ACTIONs, and LENGTH connect to each other? Metaphor is first of all a phenomenon of pragmatics. For example, when we talk about spatial metaphors, we can find metaphorical expressions such as “we grow close to (or far apart from) people,” or “one friend is at the top of the class” (e.g., Lakoff and Johnson, 1980). The experience of “close” and “top” lets us build spatial schemas for abstract concepts such as RELATIONSHIPs and REPUTATION (Boroditsky, 2000; Meier et al., 2007; Schubert et al., 2009). We can easily find a direct connection between TIME and LENGTH in our daily expressions (an hour is longer than a minute), whereas the connection between ABSTRACT ACTION and LENGTH is not so close (it took me a *long* time to finish my homework). It is not surprising that these expressions differ because “we perceive durations as being filled by particular events” (Liverence and Scholl, 2012). However, experience of ABSTRACT ACTION could be gathered and such abstract concepts could be learned during our lifetime (Chen and Zhu, 2014). In addition, it seems that ABSTRACT ACTIONs should be more closely connected to TIME than to LENGTH. Therefore, we assumed there would be direct mappings between ABSTRACT ACTIONs and TIME. Indeed, this is what we found. More importantly, by manipulating the displayed LENGTH of an event, we showed that TIME affects the accessing of ABSTRACT ACTIONs. During long-time language use, the indirectly embodied connection of ABSTRACT ACTION-TIME is built. This connection affects our word processing.

These elements might not be clear or direct, but they did build the connection between two symbolic “unrelated” concepts. These connections were a function of experience during concept learning. For example, the perception of time is part of human experience; it is essential for everyday behavior and for the survival of the individual organism (Pöppel, 1997; Buhusi and Meck, 2005; Wittmann, 2009). Then how can we “experience” that an hour is *longer* than a minute (LONG–SHORT metaphor for TIME; see Chen and Zhang, 2011)? At least we can walk a *longer* distance in an hour than in a minute. Through this experience, the concrete concept of distance becomes the connection between TIME and SPATIAL INFORMATION. Therefore, the key problem was the same under the present experimental tasks: whether there are common elements between two concepts stored in our long-term memory that can be used in accessing words. Therefore, the common element for TIME and ABSTRACT ACTIONs was the connection to LENGTH, though the strength of connection between TIME and LENGTH, and between ABSTRACT ACTION and LENGTH, might not be the same.

We claim that the “common element” could be the key to combining discussion about word processing under the amodal symbols view and discussion about accessing concepts under embodiment theory. In other words, common elements, either coming from a symbolic system or an embodied system, can facilitate word processing. More importantly, people might flexibly choose whatever is important and when the chosen element should be used. This assumption is one of the plausible explanations for some inconsistent findings under embodiment theory. Some studies have found effects of abstract domain processing on concrete domain processing; based on these results, some researchers have claimed that concrete domains are automatically activated by the situation, and then structure the abstract domains and frame the understanding and reasoning about them (Teuscher et al., 2008; Weger and Pratt, 2008; Ouellet et al., 2010). In contrast, some researchers have argued that the relational structure is stored with the abstract domains, allowing the processing of the abstract domains without having to activate the concrete domains (Santiago et al., 2011). We believe that these different findings are due to what kind of element or information was chosen by participants in the given tasks.

For example, Chinese characters used in the present study hold explicit symbolic information, such as “
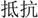
- resist”, in which “
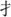
” always exists in characters that relate to actions by hands, such as “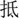-against” and “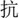-beat back.” Thus, symbolic information provided by “
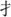
” could be strongly activated in some studies (Zhang et al., 2014a,b; Ma et al., 2015). By contrast, according to the present findings, using common elements was more effective than symbolic information under the present paradigm. Therefore, a dynamic conceptual model should be proposed: Our mental conceptual system was built up by several kinds of information, and we choose the most important or available information under the given task. Thus, the flexible accessing process should be the essence of the dynamic conceptual model.

In addition, we do not argue that mapping between abstract concepts could be as strong as that between an abstract concept and a concrete concept. Compared with the properties of priming in Experiment 2, the length-changing lines used in Experiment 1 were more concrete and their starting point and ending point were more easily captured. As a consequence, length-changing lines produced a larger difference between the consistent and inconsistent priming condition. We believe that besides perceptual information of TIME, length-changing lines might provide concrete information that is more easily activated or accessed, a conjecture that still needs more evidence to prove. Moreover, in the present dynamic system we propose, common embodied information might have less of an effect on concrete-concrete words’ mapping. For example, there are several spatial mappings (e.g., UP–DOWN, LEFT–RIGHT, and LONG–SHORT) for TIME, but the experience of TIME, which can be seen as a common element among the above mentioned concrete concepts, should not facilitate spatial–spatial (e.g., “UP–DOWN”-“LEFT–RIGHT”) concepts’ mapping. That is, a concept must first be important to another given concept, and then it becomes the common element.

## Conclusion

The more information received, including symbolic or embodied information, the more easily the concept can be accessed.

## Author Contributions

XC: Design and drafting the article. GW: analyzed data and explain the results. YL: collection and analysis of data.

## Conflict of Interest Statement

The authors declare that the research was conducted in the absence of any commercial or financial relationships that could be construed as a potential conflict of interest.
